# Prevalence of the Huschke Foramen in Colombian Population: An Important Anatomic Alteration for the Planning of TMJ Arthroscopy

**DOI:** 10.1007/s12663-024-02271-9

**Published:** 2024-08-27

**Authors:** Luis Vicente Gonzalez, Juan Pablo López, María Paula Orjuela, Michel Hernandez-Restrepo, Mitchell Calvin Balentien

**Affiliations:** 1 Oral and Maxillofacial Surgeon, Hospital Universitario La Samaritana, Bogotá, Colombia; 2 Department of Oral Research, School of Dentistry Institución,, Universitaria de Colegios de Colombia UNICOC, Bogotá, Colombia; 3 Oral and Maxillofacial Surgeon, Hospital Universitario Fundación Santa Fe de Bogotá, Bogotá, Colombia; 4 Unidad de Investigación en Epidemiología Clínica Oral UNIECLO, Universidad El Bosque, Bogotá, Colombia; 5 Scientific Coordinator of Radiology and Diagnostic Imaging, Hospital Universitario La Samaritana, Bogotá, Colombia; 6 University of La Sabana, Bogotá, Colombia; 7 Oral and Maxillofacial Resident, Universidad Pontificia La Javeriana, Bogotá, Colombia

**Keywords:** External auditory canal, Foramen tympanicum, Herniation, Temporomandibular joint

## Abstract

**Background:**

Ignoring this anatomic structure would have implications for iatrogenic perforation with the trocar toward the FH during the initial blind drilling or due to the diffusion of the infusion liquid toward the middle ear.

**Purpose:**

To analyze the prevalence of the FH in an institutional population with a high incidence of TMD to provide further guidelines in diagnosing this anomaly and the planification of TMJ arthroscopy.

**Materials and Methods:**

A retrospective tomographic study was conducted at the ENT—Oral and Maxillofacial and Radiology Department of the Hospital Universitario la Samaritana in Bogotá, Colombia. Inclusion criteria were patients over 18 years of age who had complementary exams such as ear, face, paranasal sinus, and/or TMJ tomography. Exclusion criteria were history of direct trauma to the external auditory canal in the medical history, patients with craniofacial syndromes, congenital anomalies, and/or history of cranial, mandibular, or temporal fractures. Two radiologists were part of the evaluators of the CT images who conducted the measures in the axial tomographic section and established the presence of the tympanic defect.

**Results:**

A sample size of 139 medical records of patients, where females represent *n*: 101 (72.6%) and males represent *n*: 38 (27.4%). The average age was 43 years ± 18 years. Among the studied population, a total of five FH were detected, corresponding to a prevalence of 3.6% (95% CI 1.5–8.1%). The average size of the defect was 3.52 mm ± 1.1 mm. All the patients had TMJ-related symptoms, but none of them reported otalgia.

**Conclusion:**

The initial evaluation of each patient must be addressed to assess the integrity of the tympanic bone. In this study, the defects size was smaller than others previously reported. However, diffusion through the tympanic defect could spread the lavage substance into the middle ear during TMJ arthroscopic surgery.

## Introduction

Emil Huschke described the tympanic foramen in 1889, which is why it is also known as the foramen of Huschke (FH). A developmental abnormality of the tympanic plate in the anteroinferior portion of the external auditory canal is considered, which corresponds to the posteromedial portion of the temporomandibular joint. This dehiscence generates communication between these two structures and is caused by the incomplete fusion of the tympanic plate process [1, 2]. The ossification of the external auditory canal begins with the fusion of the anterior and posterior portions of the tympanic plate. The fusion has a long process in which the foramen reduces in size and calcifies after five years, at which time its persistence is considered normal [1, 3].

The lack of studies and publications that determine the prevalence of Huschke’s foramen in the Latin American population represents a key problem for the evaluation of patients with temporomandibular disorders since the tympanic plate is an anatomic structure that directly impacts the structural integrity of the posterior portion of the temporomandibular joint (TMJ) and its relationship with the articular disk.

**Fig. 1:**
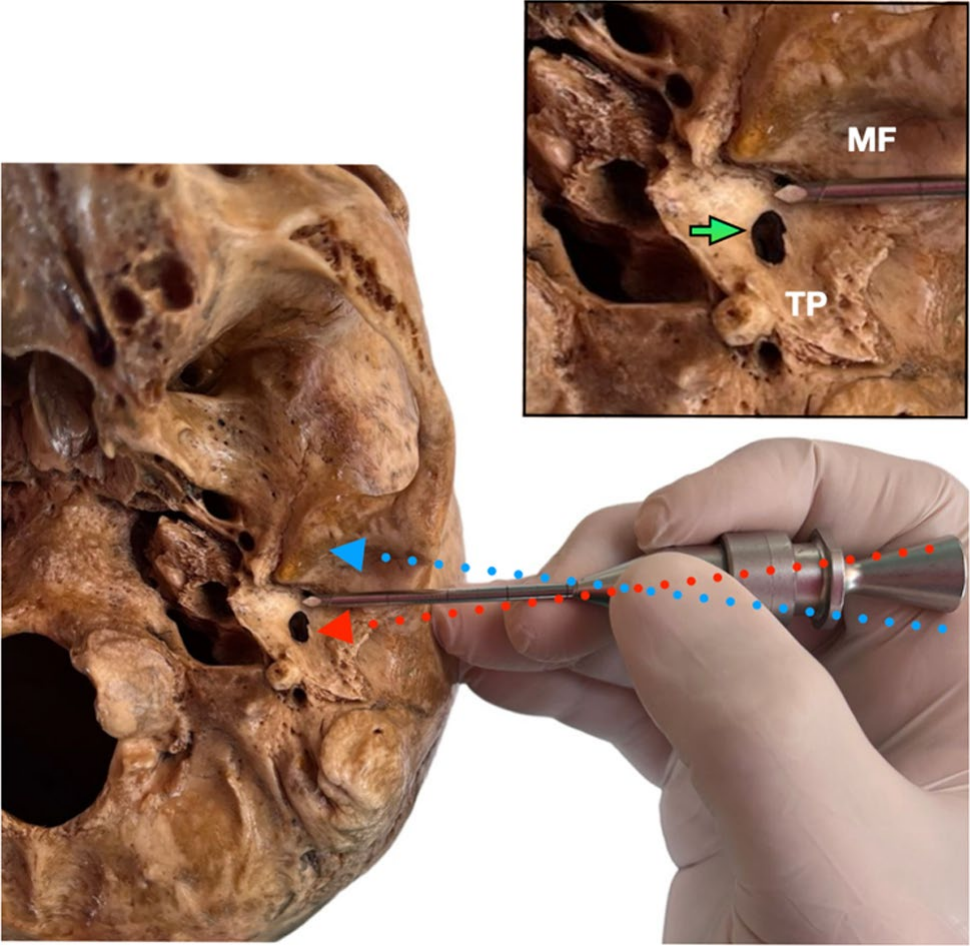
Angulation must be considered during a blind temporomandibular arthroscopy entrance with a sharp trocar. (Red dotted arrow) Posterior dangerous angulation must be avoided in order not to damage the integrity of the tympanic defect. (Blue dotted arrow) Anterior safe angulation to avoid the tympanic plate. During the TMJ arthroscopy, the security deep marks must be cheeked. No more than 25 mm must be the arthroscope introduced. Green arrow: marks deep control check from the patient skin. White arrow: the tip of the arthroscope close to the tympanic defect could increase the pressure and lead to an irrigation spread to the middle ear. Abbreviations. TP: Tympanic plate. MF: Mandibular fossa

**Fig. 2:**
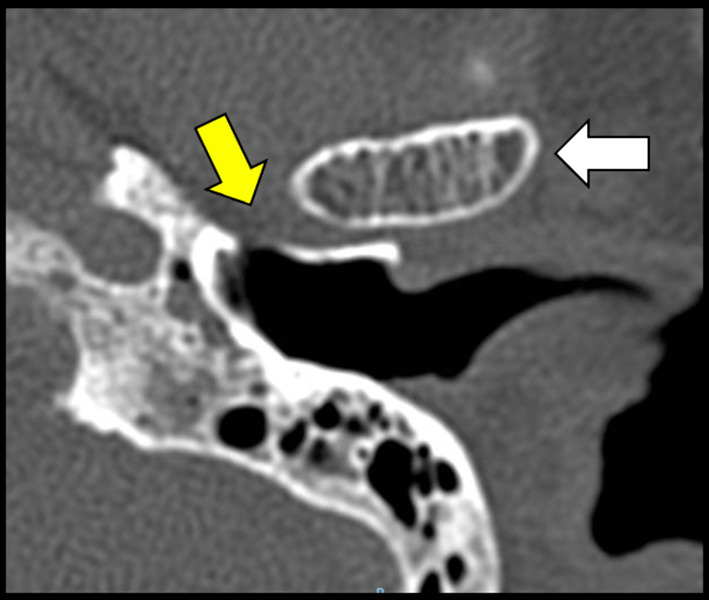
CT Axial view. Tympanic defect (yellow arrow) Mandibular condyle (white arrow)

The TMJ arthroscopy is a minimally invasive technique with a valuable approach to the management of temporomandibular disorders (TMD). An MRI should be assessed during the presurgical evaluation to establish the disk position and the tympanic area integrity. The tympanic dehiscence can be the cause of multiple issues, concerning not only the patient when a possible herniation of retrodiscal tissues exists into the external auditory canal [4], which can be asymptomatic but also surgeons performing TMJ arthroscopy because of the possibility of perforating the adjacent tissues. During the first step of the TMJ arthroscopy, to the entrance to the upper joint space, the maneuver is totally blind with a sharp and blunt trocar, which is a dangerous angulation (Fig. 1) and deep (Fig. 2) must be considered to about tympanic damage. Thus, the present study analyzes the prevalence of the FH in an institutional population with a high incidence of TMD to provide further guidelines in diagnosing this anomaly and planning TMJ arthroscopy.

## Materials and Methods

A retrospective tomographic study was conducted at the ENT—Oral and Maxillofacial and Radiology Department of the Hospital Universitario la Samaritana in Bogotá, Colombia*.* A group of patients who consulted for TMD from 2019 to 2022 was evaluated under the institution's previous authorization of the ethical committee (#07-2020). Medical records were filtered with the following selection criteria: adequately filled medical records of patients over 18 years of age with complementary exams such as ear, face, paranasal sinus, and/or TMJ tomography. Exclusion criteria were a history of direct trauma to the external auditory canal in the medical history, patients with craniofacial syndromes, congenital anomalies, and/or history of cranial, mandibular, or temporal fractures. Two radiologists were part of the evaluators of the CT images who conducted the measures in the axial tomographic section and established the presence of the tympanic defect.

Computers with the “AW advantage workstation volume share 3D and 4D applications” were used for data analysis. The tomographic images were obtained with the CT multislice equipment of the general electric brand, model lightspeed VCT. A radiologist of the institution read and analyzed the scans for defect identification by a previously calibrated examiner (Kappa 0.92) to identify the presence or absence of FH with its respective size in the axial plane and laterality. To measure and identify the foramina for every patient, we noted the presence of a foramen, its size, and its location in relation to the tympanic membrane. We measured its distance to the anterior insertion of the tympanic membrane to determine its precise location [5]. Measurements of prevalence ratios with 95% CI were estimated. Quantitative and qualitative variables were compared using t test/u-MANN–WHITNEY accompanied by a bivariate analysis performed using X2/Fisher’s exact test for frequency comparison. All data were analyzed using IBM SPSS software.

## Results

A sample size of 139 medical records of patients, where females represent *n*: 101 (72.6%) and males represent *n*: 38 (27.4%). The average age was 43 years ± 18 years. Overall, 42.4% of patients (95% CI 34.5–50.8%) were born in Bogota, 43.2% (95% CI 35.2–51.5%) of patients were from other cities in the state of Cundinamarca, and the rest of patients were from other cities in Colombia (14.4–95% CI 9.5–21.2%).

Regarding the diagnostic images studied, 8.6% (95% CI 5–14.5%) corresponded to ear, petrous bone, and internal auditory canal tomographies, and 91.4% (95% CI 85.5–95%) were paranasal sinuses/face tomographies (Fig. 2).

Among the studied population, a total of five FH (female patients) were detected, corresponding to a prevalence of 3.6% (95% CI 1.5–8.1%). The average size of the defect was 3.52 mm ± 1.1 mm (Table 1). All the patients had TMJ-related symptoms (pain), but none of them reported otalgia or any symptom associated with the otologic component.

**Table 1:** Characteristics of each FH detected

Defect	Laterality	Dimensions (mm)
FH 1	Left	4.16
FH 2	Left	4.66
FH 3	Right	1.87
FH 4	Right	2.99
FH 5	Right	3.90

## Discussion

The TMJ herniation into the external auditory canal (EAC) is a rare condition reported in the literature [6, 7]. TMJ Pain is a common symptom mentioned in some cases of HF [8]. In this study, the population considered were patients with TMD. No relationship was found between the TMJ and otologic symptomatology with the presence of timpani defect. This could have been related to the defect size average of 3.52 mm ± 1.1 mm found in this study. According to the study by Park et al. [9], the size of the defect is a determinant factor for the presence of symptoms. They reported that the mean size of the anterior wall defects in the studied group of TMJ herniation patients was 6.17 × 5.33 mm. The incidence of FH was 3.6% in our tomographic study. These results are relatively low compared to other published studies that reported a prevalence between 1.5 and 22.7% in tomography evaluation [10, 11] and an osteological study report that showed a prevalence of 7.2% [12].

An important factor should be considered to establish a geographical distribution of this condition: the membranous ossification patterns differences between races. Thus, race might affect the incidence of FH [13]. A meta-analysis established the highest prevalence of FH in Asia (21.4%) [14]. Compared with other studies that reported the incidence of FH in different countries, the Netherlands showed 4.6% [5], Turkey 13.4% [2], and Brazil 12.7% [6]. The literature poorly reveals HF prevalence in Europe, North, and South America. For this reason, future researchers may consider tomographic and anatomic studies to establish a solid prevalence in the different continents.

One relevant clinical issue is that this tympanic defect could be repaired in patients with a severe symptom like otalgia, or the FH could be part of a fistula of salivary otorrhea [15]; the literature reports that 50% of the cases reporting FH were under surgical correction [16], and different sorts of techniques are being described, including endoscopic-assisted transcanal repair of the tympanic defects [17], the use of polyethylene plates through a preauricular approach for the reconstruction of the tympanic defect [18], or auricle cartilage [19].

On the other hand, this anatomic defect could be asymptomatic in patients with TMD; for this reason, during the assessment of a TMD, patients must be at considerable risk of otologic injury during a TMJ arthroscopy procedure [20]. This study represents a group of patients with TMD who have a low incidence of this anatomic defect. Despite the literature reporting a low incidence of complications during the TMJ arthroscopy [21, 22], there are cases reported related to otologic complications, including tympanic membrane rupture, dislocation of the incus, injury to the tympanic segment of the facial nerve, labyrinthine disruption, and ear infection [23].

The relevant consideration of this defect should be set as an early fundamental issue during the training in TMJ arthroscopy due to the learning curve. The most common mistake is the angulation during the blind entrance to the upper joint space; a minimal angulation variation over 15°in the axial plane could lead to otologic damage [24]. The presence of FH in a patient who will undergo arthroscopy procedures could increase the risk of intraoperative complications. This is mainly due to two situations. The first is that during blind drilling with the acute trocar, if there is an incorrect inclination with a slight posterior direction, it could lead to a perforation of the membrane and facilitate entry into the middle ear. The second is that if the membrane was injured but not perforated and the fluid entry pressure or the depth of the arthroscopy is not controlled, the diffusion of the irrigation fluid can be facilitated, and otic pathology may present in the postoperative period. Thus, the initial evaluation of each patient must be addressed to assess the integrity of the tympanic bone.

The literature describes no relevant studies about this consideration, and currently, the surgical minimally invasive approach of TMD is becoming the first treatment option. During the arthroscopic procedure, the rigid instruments are placed in the posterior medial area of the mandibular fossa, and the risk of perforation, infiltration, and effusion of the irrigation solution could spread into the middle ear and lead to hearing impairment. In this study, the defect size was smaller than others previously reported. However, diffusion through the tympanic defect could spread the lavage substance into the middle ear during an arthroscopic procedure. In our experience, the prevalence of HF in TMD patients was low. Nevertheless, establishing a real prevalence in the population is very important, which is why other studies should be accomplished in different patient groups. More studies are required to establish a real prevalence of complications during the arthroscopic procedure in patients with TMD and FH which represent an ideal group of study.
